# Aging through the time of COVID-19: a survey of self-reported healthcare access

**DOI:** 10.1186/s12913-021-07353-9

**Published:** 2021-12-19

**Authors:** Allie Peckham, Keenan A. Pituch, Molly Maxfield, M. Aaron Guest, Shalini Sivanandam, Bradley N. Doebbeling

**Affiliations:** 1 Center for Innovation in Healthy and Resilient Aging, Arizona State University, 550 North 3rd St, Phoenix, AZ 85004 USA; 2 Edson College of Nursing and Health Innovation, Arizona State University, 550 North 3rd St, Phoenix, AZ 85004 USA; 3 North American Observatory on Health Systems and Policies, University of Toronto, 155 College St, Toronto, ON Canada; 4 College of Health Solutions, Arizona State University, 500 North 3rd St, Phoenix, AZ 85004 USA

**Keywords:** COVID-19, Chronic conditions, Healthcare access, Pandemic, Barriers, Older adults

## Abstract

**Background:**

Chronic conditions are common and require ongoing continuous management and preventive measures. The COVID-19 pandemic may have affected the management of chronic conditions by delaying care. We sought to understand the impact of personal characteristics (i.e., age) and healthcare factors (i.e., access to a provider) on healthcare access in a sample of Americans 50 years of age or older during COVID-19.

**Method:**

Participants completed an online survey at the start of the COVID-19 pandemic – the Aging in the Time of COVID Survey. Questions focused on health status, health care access, COVID-19 fear, and social connectedness. Participants were recruited through social media advertisements, list serves, and snowball sampling. Data collection started in early April 2020 and concluded in late May 2020. Logistic regression models examined the results of two key access points: healthcare provider/doctor (*n* = 481) and medication (*n* = 765), with 56 and 93% of participants reporting access to a provider and medications, respectively.

**Results:**

Individuals with an established primary care provider were much more likely to obtain access to a healthcare provider, *OR* = 3.81 (95% CI: 1.69, 8.77), and to receive medication, *OR* = 4.48 (95% CI: 1.61, 11.48), during the time of COVID-19. In addition, access to medication was (a) higher for those who were older, *OR* = 1.05 (95% CI: 1.01, 1.09), had a higher income (greater than 100 k compared to less than 50 k, *OR* = 3.04 (95% CI: 1.11, 8.98), and (b) lower for those having caregiving responsibilities, *OR* = 0.41 (95% CI: 0.21, 0.78), or greater social isolation, *OR* = 0.93 (95% CI: 0.87, 0.98).

**Conclusions:**

Although most participants had access to medication, just over half had access to a healthcare provider when needed. Notably, health-seeking behaviors for individuals who do not have an established primary care providers as well as those who provide unpaid care, are socially isolated, and younger may require more proactive approaches to care monitoring, management, and maintenance.

## Background

Over 80% of adults aged 65 and older in the U.S. have multiple chronic conditions (MCC), which include depression, diabetes, heart disease, or dementia [1, 2]. Primary care has become increasingly important for the on-going management of chronic conditions that require preventative treatment, regular follow-ups, and management of multiple medications [3]. This management of chronic conditions is critically important to reduce mortality, maintain functional status and quality of life of older adults, and sustain or reduce health system costs. These routine visits work to prevent illness and limit chronic disease burden [1, 2, 4–7].

Many healthcare providers have voiced concern about the adverse short and long-term outcomes caused by delay or avoidance of accessing healthcare during the COVID-19 pandemic [8–10]. These concerns come from an understanding that poor management of chronic conditions can lead to additional health issues, worsening of the primary problem and can increase the likelihood of an acute episode leading to an otherwise avoidable hospitalization or premature death [3, 11–13].

Timely and appropriate access to healthcare providers and medications is important for monitoring, preventing, and maintaining overall health and well-being. In fact, limited or no access can have major longer-term impacts on disease incidence, exacerbations, and complications [3–5, 11–13].

In this particular research, we sought to understand healthcare access (provider and medications) during COVID-19 in a sample of adults 50 years of age and older residing in the United States. This research is a part of a larger web-based study examining health and well-being among mid-life and older adults. This is a developing area of research where knowledge identifying the influence that COVID-19 has on healthcare access and management of chronic conditions is only beginning to emerge. Some have focused on the views from health care professionals that suggest routine care and management of hypertension, diabetes, and chronic obstructive pulmonary disease were the most impacted condition at the onset of the pandemic [9]. Others assessed the impact of telemedicine use on outpatient care visits using claims data [14] and the benefits and barriers to virtual care for managing chronic conditions [15].

The profusion of stay-at-home orders and efforts to reduce in-person close contact have also led to shifts in how healthcare is being delivered. Healthcare providers and patients have prioritized urgent visits and minimized in-person contact [16]. Given this, we anticipate individuals may experience less access (to healthcare providers and medications) during COVID-19. We pay particular attention to those living with chronic conditions, given the importance of on-going management for this population and potential longer-term impactions of lack of access.

## Methods

The data were collected as a component to the larger ‘Aging in the Time of COVID-19’ study, a longitudinal, web based multi-wave study, conducted in 2020. Participants were recruited through online advertisements on social media platforms (Twitter, Facebook) posted by the Center for Innovation in Healthy and Resilient Aging, online list-serves, and university forums. Individuals were eligible to participate if they were aged 50 years or older and were English speaking. Given the English nature of the survey, and to minimize the variability in healthcare plans and policy responses from other countries, for this study, we focus on individuals who reside in the United States and completed the first wave of data collection. We included adults aged 50 and older to examine access issues specific to late midlife and older adulthood. Although the number of such survey respondents was 1443, we focus our study on a subset of these cases, as described later. Given rapid changes in COVID-19 policies occurring at the time, the present research was conceptualized and implemented within a 1.5 week span. This final survey did not undergo pilot or validity testing, but the assessments included were either previously validated or based on previously validated measures, with minor adjustments to increase relevance during COVID. The online survey included questions about a variety of experiences during the time of COVID-19. Data collection for the reported findings was completed using REDCap and ran from April 13–May 15th, 2020 [17]. The information was collected during the height of the stay-at-home orders in the United States. Following completion of the survey, participants had the option to be entered into a raffle to win one of five $25.00 gift cards. The study was approved by the Arizona State University IRB. The methods have been described more fully elsewhere [18, 19].

After agreeing to the informed consent, participants were asked basic demographic questions. Participants were also asked to indicate the presence or absence of chronic conditions. Using items from the 2017 Behavioral Risk Factor Surveillance Survey (BRFSS) Disease Scale [20]. Additionally, participants were asked about healthcare access related to several key access points (housing, transportation, support groups, legal services, dental and vision services, in-home health services, occupational and physical therapy, healthcare provider/doctor, medication, emergency room, counseling/therapy, and food assistance). Specifically, “We would like to ask you about services you may or may not need and may or may not have been able to obtain in the last two weeks due to COVID-19. Thinking about the services listed, please check the box that is appropriate.” Participants could select one of four options: needed and received, needed and did not receive because of COVID-19, needed and did not receive due to reasons other than COVID-19, and did not need. We focused on access to a healthcare provider and medication as these two access points are seen as important for the management of chronic conditions. These were considered the most representative of the access AHRQ access dimensions (e.g., coverage, services, timeliness and workforce), available on the survey. These measures are not exclusive nor exhaustive, but two important points to consider [21]. In addition, we eliminated 962 cases that responded not needing access to a healthcare provider and 678 who reported not needing access to medications because we were interested in those who needed such access. As such, the analytic sample size for the healthcare provider outcome was 481 and 765 for the medication’s outcome.

Demographic and health-related variables were collected for this study. The demographic variables included age (numeric, with ages ranging from 50 to 87), sex (1 = female, 0 = male), race (1 = White, 0 = Other), educational status (represented by two-dummy coded predictors, with those having not attained a Bachelors’ degree serving as the reference group and those with a Bachelor’s degree and those with a graduate degree comprising the other two groups), employment status [1 = employed (full or part-time or self-employed), 0 = not employed (unemployed, homemaker, student, retired, unable to work)], relationship status [1 = partnered (married or member of an unmarried couple), 0 = other], total annual household income (represented by two dummy-coded predictors with income < $50,000 serving as the reference group and those with income between $50,000 and $100,000 and then income > $100,000 comprising the other two groups), and caregiving responsibility (1 = yes, 0 = no). Note that with the exception of age, all of the demographic variables were categorical.

Several health-related predictors were collected and included the study. Participant self-rated health (numeric) was assessed through the single item health question, where participants were asked, “In general, how would you rate your health today?” Responses options were: 1 = very good, 2 = good, 3 = moderate, 4 = bad, and 5 = very bad. Scores were reverse coded for all analyses such that higher scores were indicative of better health (i.e., 1 = very bad and 5 = very good health) [22]. Other health-related predictors include the sum of the chronic health conditions indicated (numeric, with scores ranging from 0 to 10), UCLA loneliness (numeric, with scores ranging from 25 to 79 out of a possible 20 to 80, with higher scores equated to increased loneliness.), [23] PROMIS social isolation (numeric, with scores ranging from 34.8 to 74.2 out of a possible 34.8 to 74.2 with a population average of 50, and scores over 50 indicating higher rates of social isolation), and primary care provider status (1 = has a primary care provider; 0 = otherwise) [24].

### Statistical analysis

We first examined basic descriptive statistics and frequencies to identify if unusual values were present and determine the extent of incomplete data. No unusual scores were present for a given variable, but participants had missing data for most [11] of the variables, including the outcomes, where 13 participants had missing data for provider access and 11 had missing data for medication access. Although the percentage of missing data did not exceed 4% for any given variable (with this missingness rate occurring for age and income), dropping cases with missing data (as listwise deletion does) would have resulted in removing up to 14% of the participants from the analyses, resulting in potential estimation bias and loss of power. As such, for the primary analysis, we used a modern missing data treatment, as described below, that effectively treats missing data for predictor and outcome variables. We also examined index plots (i.e., Mahalanobis’ distance by case number) to assess if multivariate outliers were present and examined values of the variance inflation factor to assess if multicollinearity was present. Although no troublesome multicollinearity was present (each variance inflation factor < 3), a multivariate outlier was detected. Inclusion or exclusion of this case did not alter any study conclusion. As such, this case was included in all analyses.

To examine associations between the demographic and health-related predictors (i.e., chronic health conditions, self-perceived health, loneliness, social isolation, established relationship with a primary care provider) and the access outcomes (i.e., access to a healthcare provider and access to medications), we first computed descriptive statistics to describe these variables by each of the outcome categories, as well as conducting bivariate statistical tests (i.e., independent samples *t* tests and chi-square tests of association). For the primary analysis, we estimated a logistic regression model for each outcome while simultaneously treating missing data on the outcomes and predictors. For this purpose, we obtained model parameters using Bayesian Markov Chain Monte Carlo (MCMC) estimation, which provides unbiased parameter estimates when data are missing at random [25, 26], with this missing data treatment also resulting in retaining in the analysis each participant who reported data for any study variable. Analogous to Firth estimation procedures [27], Bayesian estimation is also recommended for logistic regression when sparse data are present, because such estimation can remove bias in the estimates of regression coefficients and their standard errors [28, 29]. Note, though, that to avoid potential estimation problems that could arise due to a very small number of events, we combined the two “not received” categories due to the small number of participants who responded they did not receive access due to reasons other than COVID-19 (*n* = 28 and *n =* 23 for the access to a health provider and medication outcomes, respectively). Thus, for the logistic regression models, the two outcome categories were received or did not receive access. This analysis was implemented in Mplus software, Version 8.6 [30], using weakly informative prior distributions for the regression coefficients, as recommended for logistic regression [28, 29]. We monitored model convergence with the potential scale reduction factor, with a value less than 1.10 indicating convergence. When we estimated the models, the maximum potential scale reduction factor obtained was smaller than 1.01 for all parameter estimates for each of the iterations used to obtain parameter estimates, indicative of superior estimation.

Unlike traditional analyses, Bayesian estimation produces a distribution of values for each model parameter, and we requested 10,000 random draws to build these posterior distributions (after 10,000 burn-iterations). The median of these posterior distributions was used to represent final parameter estimates (i.e., logistic regression coefficients). Further, although we report two-sided *p*-values associated with the regression coefficients, the 2.5th and 97.5th values from the posterior distributions were used to form 95% Bayesian highest density credible intervals, which, when not containing a value of zero, is comparable to achieving statistical significance in traditional analyses with an alpha level equal to 0.05. Note that, analogous to bootstrapping, the use of such intervals does not rely on distributional assumptions or large-sample theory. To convey the practical importance, or meaningfulness, of the analysis results we computed and graphed model estimated probabilities of access for significant predictors, with the estimated probabilities and graphs obtained via SAS® software, version 9.4 M7 [31].

## Results

### Participant characteristics by outcome status

Table 1 presents descriptive statistics and associated test results for the demographic and health-related study variables shown by category for the two study outcomes (access to a health provider and medications). Of the 468 participants who provided complete data for the healthcare provider outcome and reported needing access, 263 (56%) indicated they received access to a healthcare provider. Compared to those who reported not receiving healthcare provider access, participants who indicated receiving such access were of older age, on average, (63.7 vs. 61.7, *p =* .006) and had greater mean scores on self-perceived health (4.0 vs. 3.8, *p* = .02), but lower means on UCLA loneliness (48.6 vs. 52.1, *p =* .00003) and PROMIS social isolation (49.6 vs. 52.8, *p* = .0001). In addition, a greater proportion of participants having an established primary care provider were more likely to obtain access to a health care provider than those without a primary care provider (58.7% vs. 25.7%, *p =* .0002). Of the 754 participants who provided complete data for the medications outcome and indicated they needed such access, 703 (93%) reported they received access. Compared to those not receiving access to medications, participants who reported receiving such access were, on average, of older age (63.6 vs. 61.2, *p* = .03) and had lower mean scores on UCLA loneliness (48.8 vs. 52.1, *p* = .01) and PROMIS social isolation (49.5 vs. 54.3, *p* = .0002). In addition, participants who did not have caregiving responsibilities were more likely to obtain access to medications than those with such responsibilities (95.5% vs. 90.7%, *p =* .009), as were participants with a primary care provider than without (94.0% vs. 77.1%, *p* = .0001) and those who reported greater annual household income (*p =* .005), particularly participants reporting an income greater than 100 K compared to those with an income of less than 50 K (96.5% vs. 88.7%, *p* = .006).

**Table 1:** Descriptive Statistics for Demographic and Health-Related Variables by Outcome and Access Status

Variable	Access to health care provider			Access to medications		
	Received (*n* = 263)	Not received (*n* = 205)		Received (*n* = 703)	Not received (*n* = 51)	
	*M ± SD* or *n* (%)	*M ± SD* or *n* (%)	*P*^a^	*M ± SD* or *n* (%)	*M ± SD* or *n* (%)	*P*^a^
Age	63.66 ± 8.13	61.66 ± 6.89	.006	63.60 ± 7.60	61.22 ± 7.47	.03
Sex			.15			.39
Female	228 (54.9)	187 (45.1)		618 (92.9)	47 (7.1)	
Male	34 (65.4)	18 (34.6)		83 (95.4)	4 (4.6)	
Race			.37			.97
White	245 (55.7)	195 (44.3)		667 (93.2)	49 (6.8)	
Other	15 (65.2)	8 (34.8)		28 (93.3)	2 (6.7)	
Education			.20			.34
Some college or less	58 (55.2)	47 (44.8)		169 (90.9)	17 (9.1)	
College graduate	76 (50.7)	74 (49.3)		209 (93.3)	15 (6.7)	
Advanced degree	124 (60.2)	82 (39.8)		313 (94.3)	19 (5.7)	
Employed			.35			.41
Yes	115 (53.7)	99 (46.3)		300 (94.0)	19 (6.0)	
No	144 (58.1)	104 (41.9)		394 (92.5)	32 (7.5)	
Married (or unmarried) couple			.26			.47
Yes	168 (58.1)	121 (41.9)		448 (93.7)	30 (6.3)	
No	94 (52.8)	84 (47.2)		254 (92.4)	21 (7.6)	
Annual household income			.17			.005
< 50 K	67 (50.0)	67 (50.0)		181 (88.7)	23 (11.3)	
50 K < 100 K	98 (58.3)	70 (41.7)		278 (93.9)	18 (6.1)	
> 100 K	89 (60.5)	58 (39.5)		218 (96.5)	8 (3.5)	
Has caregiving responsibilities			.45			.009
Yes	128 (54.5)	107 (45.5)		321 (90.7)	33 (9.3)	
No	135 (57.9)	98 (42.1)		382 (95.5)	18 (4.5)	
Health related variables						
Self-perceived health	3.95 ± 0.76	3.78 ± 0.76	.02	3.98 ± 0.76	3.75 ± 0.91	.08
Sum of chronic health conditions	2.74 ± 1.87	2.54 ± 1.91	.25	2.63 ± 1.68	2.96 ± 2.13	.28
UCLA loneliness	48.63 ± 8.86	52.14 ± 9.07	.00003	48.84 ± 8.94	52.10 ± 9.97	.01
PROMIS social isolation	49.62 ± 8.92	52.77 ± 8.45	.0001	49.49 ± 8.85	54.27 ± 8.51	.0002
Has a primary care provider			.0002			.0001
Yes	253 (58.7)	178 (41.3)		672 (94.0)	43 (6.0)	
No	9 (25.7)	26 (74.3)		27 (77.1)	8 (22.9)	

*Note.* The response options for self-perceived health range from 1 = *very bad* to 5 = *very good*. The observed scores ranged from 50 to 87 for age, 0 to 10 for sum of chronic conditions, 25 to 79 for UCLA loneliness, and 34.8 to 74.2 for PROMIS social isolation

^a^Is the *p* value for the independent-samples *t* test for numeric variables and the chi-square test of association for categorical variables, each assessing differences between the two outcome categories for each demographic and health-related variable

### Predictors of access to a healthcare provider and medications

**Fig. 1:**
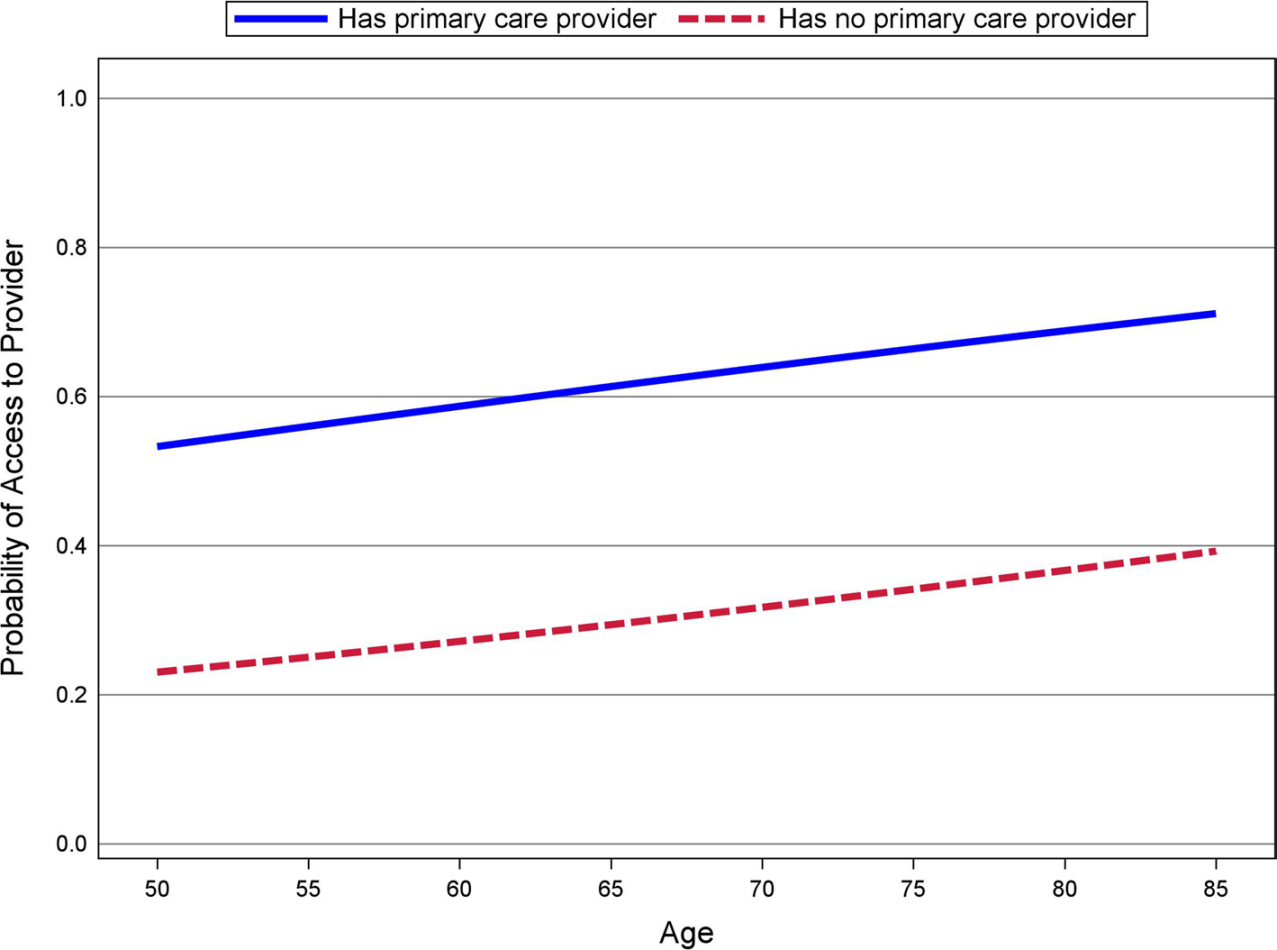
Probability of Access to Provider

Table 2 shows results of the logistic regression analyses for each outcome. For access to a healthcare provider, the only significant predictor was whether a participant had an established primary care provider (*p =* .001). The corresponding odds ratio indicates that the odds of receiving vs. not receiving access to a health provider was 3.8 times greater for participants who had an established primary care provider compared to those who did not. Figure 1 shows the model estimated probabilities of access to a healthcare provider for those who had and did not have an established primary care provider across the participant ages of 50 to 85, holding all numeric predictors at their mean value and all categorical variables at their most common value. Consistent with the percentages shown in Table 1, Fig. 1 shows that participants who had an established primary care provider were more than twice as likely to indicate that they received access to a health provider compared to participants without an established primary care provider.

**Table 2:** Logistic Regression Results for the Probability of Receiving Access to a Health Provider and Medications

Predictor	*b (SE)*	*p*	*OR*	*OR* 95% CI	*b (SE)*	*p*	*OR*^*a*^	*OR* 95% CI
	Access to provider				Access to medications			
Age	.02 (.01)	.12	1.02	[0.99, 1.05]	.05^*^ (.02)	.02	1.05^*^	[1.01, 1.09]
Female	−.25 (.33)	.45	0.78	[0.41, 1.49]	−.26 (.56)	.63	0.77	[0.25, 2.19]
White	−.45 (.48)	.34	0.64	[0.25, 1.69]	−.33 (.79)	.65	0.72	[0.15, 3.13]
Education								
Bachelor’s vs. < Bachelor’s degree	−.35 (.28)	.22	0.71	[0.41, 1.21]	.21 (.41)	.59	1.24	[0.57, 2.77]
Advanced degree vs. < Bachelor’s degree	.02 (.28)	.95	1.02	[0.59, 1.74]	.30 (.40)	.45	1.36	[0.61, 2.94]
Employed	−.11 (.23)	.63	0.90	[0.57, 1.38]	.24 (.35)	.46	1.28	[0.65, 2.54]
Couple	−.22 (.25)	.39	0.80	[0.49, 1.30]	−.05 (.38)	.89	0.95	[0.45, 1.98]
Annual household income								
50 K to 100 k vs. < 50 K	.37 (.27)	.18	1.45	[0.87, 2.54]	.59(.39)	.17	1.80	[0.84, 3.86]
100 K+ vs. < 50 K	.44 (.32)	.17	1.55	[0.84, 2.90]	1.11^*^ (.53)	.03	3.04^*^	[1.11, 8.98]
Caregiver	−.13 (.21)	.53	0.88	[0.57, 1.31]	−.90^**^ (.34)	.008	0.41^**^	[0.21, 0.78]
Self-perceived health	.20 (.15)	.17	1.22	[0.92, 1.62]	.12 (.21)	.58	1.12	[0.74, 1.69]
Sum of chronic disease conditions	.08 (.06)	.18	1.09	[0.97, 1.23]	−.09 (.09)	.33	0.91	[0.76, 1.09]
UCLA loneliness	−.03 (.02)	.16	0.98	[0.94, 1.01]	.04 (.03)	.16	1.04	[0.98, 1.10]
PROMIS social isolation	−.02 (.02)	.23	0.98	[0.94, 1.02]	−.08^**^ (.03)	.008	0.93^**^	[0.87, 0.98]
Primary care provider	1.34^**^ (.42)	.002	3.81^**^	[1.69, 8.77]	1.50^**^ (.50)	.004	4.48^**^	[1.61, 11.48]
Intercept	−.16 (1.31)				.62			

*Note.*Female is coded as 1 = female, 0 = male. White is coded as 1 = White, 0 = other. Employed is coded as 1 = employed, 0 = unemployed. Couple is coded as 1 = married or unmarried couple, 0 = otherwise. Caregiver is coded as 1 = participant has caregiving responsibilities, 0 = otherwise. Primary care provider is coded as 1 = participant has a primary care provider, 0 = otherwise. *b (SE)* is a logistic regression coefficient (and standard error), *p* is a two-tailed *p* value (obtained by multiplying the one-tailed p-value output by Mplus by 2), and *OR* is the corresponding odds ratio

^*^95% Bayesian highest density credible interval for the regression coefficient does not include 0

^**^99% Bayesian highest density credible interval for the regression coefficient does not include 0

For access to medications, the results in Table 2 indicate that having an established primary care provider (*p =* .002) was strongly related to such access, with the odds of receiving access to medications being approximately 4.5 times greater for those with than without an established primary care provider. In addition, four other predictors were related to medication access. Specifically, greater access to medications was reported by participants who were older (*p =* .008), had an income greater than $100 K annually, compared to those with an income below $50 K (*p =* .016), did not have caregiving responsibilities (*p* = .004), and had lower social isolation scores (*p* = .004).

**Fig. 2:**
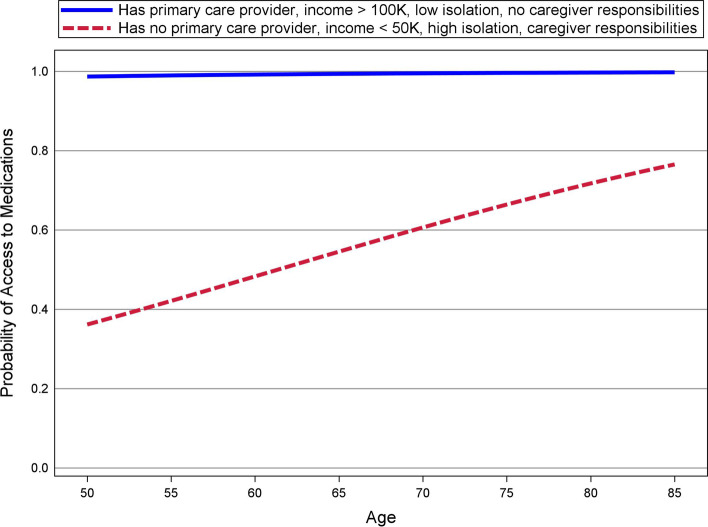
Probability of Access to Medication

Figure 2 shows the model estimated probabilities of access to medications for two profiles of participants based on the significant predictors. The first profile comprises those who had an established primary care provider, a household annual income > $100 K, a social isolation score that is 1 standard deviation below the mean, and no caregiving responsibilities. The other profile comprises those who had no established primary care provider, a household income < $50 K annually, a social isolation score that was 1 standard deviation above the mean, and caregiving responsibilities. The other predictors that were not related to medication access were held constant at their mean (for numeric variables) or most common value for the categorical variables. Figure 2 shows that the likelihood of receiving access to medications, computed across the ages of 50 to 85, was virtually 1 for the first profile, whereas the probability of receiving such access was strikingly lower for the second profile across the age range, but particularly for younger participants, where such estimated probabilities dropped below 0.40.

## Discussion

More chronic conditions did not interfere with access in this population. What seemed particularly important was having a pre-existing relationship with a primary healthcare provider. Our findings suggest that receiving access to a healthcare provider and medication during the time of COVID-19 was greatly associated with having a pre-existing relationship with a primary care provider, with the odds of receiving access to healthcare provider and medication, respectively, being 3.8 and 4.5 times greater for those having an established primary care provider relationship. Participants who received access were generally healthier, older, and reported greater income and less loneliness and isolation. Access to medication was also positively associated with no caregiving responsibilities. Established primary care relationships are important during times of ‘normalcy’, but are just as important if not more important for times of a pandemic, given how critical this access is to the management of chronic conditions [7, 12, 32]. Timely and appropriate access to primary care to manage chronic conditions can prevent complications or exacerbations that may lead to more costly and inappropriate service use. Those individuals without pre-established healthcare relationships would be at even greater risk to experience these complications [7, 8, 11, 12].

Social isolation has been associated with a higher risk of chronic health conditions, including, higher blood pressure, obesity, depression, and dementia [33]. The findings of this research highlight another example of the potential implications of social isolation and loneliness; they might affect an individual’s willingness and/or ability to seek primary care [34–36]. While evidence in this area is only beginning to emerge, there is research that has identified those living with chronic isolation or loneliness rely less on preventative health services [37]. Furthermore, for individuals who have chronic conditions, it could limit their treatment, leading to poorer health outcomes, and longer-term personal and health system implications [38].

We attribute high rates of access to the higher socioeconomic and education status of the sample. Most participants did have access to care when needed, particularly to medication, though not everyone had access, when needed. These findings raise concern for populations who may have poorer social determinants of health and may experience barriers to access (i.e., finances, insurance, transportation, and timeliness). Access problems are likely to be even greater among historically underserved populations (i.e., people of color, individuals with lower socioeconomic status, those living with complex multimorbidities, those with limited health insurance, LGBTQ populations, and unpaid caregivers). Social inequalities and limited social determinants of health contribute to higher incidences of these chronic conditions. Indeed, social inequalities and limited social determinants of health contribute to higher incidences of chronic conditions [39, 40]. For example, Hispanics and Black Americans are less likely to have health insurance compared to non-Hispanic Whites [41]. Lack of healthcare access can lead to undiagnosed chronic conditions and poor management of chronic conditions, which further puts these populations at risk during times of public health crises [7, 36, 41–44].

### Limitations

The limitations of the study include the use of self-reported measures and the distribution of the survey was limited to the beginning of the pandemic. It would have been ideal to continue to follow participants through the pandemic to understand how access to healthcare services changed over time. We followed this group for 3 months, and our last data collection was still in the early stages of the pandemic. It may be that access to services improved in later stages of the pandemic, as health systems, providers and patients adapted to new clinic processes and use of telemedicine when possible. The homogeneity of the sample (overrepresented by those who are White, women, middle-upper class, education, access to internet and computer, computer literate) is an important limitation. The sample is not representative of the population. However, understanding the implications of this sample with respect to healthcare access and established primary care relationships is important, and we suggest thatit is reasonable to assume that more racially and ethnically diverse populations would report greater difficulty accessing healthcare services and would be less likely to report having a primary care provider. Another limitation is that we did not ask about health insurance coverage specifically. Lack of health insurance is a barrier to access and may have contributed to age-related findings. Specifically, residents of the United States aged 65 and older are eligible for Medicare coverage, which may be why access to healthcare providers and medications was greater with older age. However, without data concerning health insurance coverage, we cannot be certain of its impact on the present findings. Additionally, the directionality for social isolation and negative outcomes (access and health) remains a question of interest that our dataset was unable to answer. Lastly, within the United States, there was significant variability in recommendations for physical distancing, ranging from strict stay-at-home orders to more understated responses to the pandemic. It would be helpful to examine how policy differences among the states influenced access to services; however, our sample was not evenly distributed amongst regions/states, which prevents this type of analysis.

## Conclusion

In conclusion, access to a provider and medication during the time of COVID-19 was strongly related to having pre-existing established relationship with a primary care provider. Those who were generally healthier, older, and reported greater income and less loneliness and isolation also had greater access. Healthcare delivery systems and providers must consider populations who may experience barriers to access during times of pandemics and normalcy. For example, understanding that health seeking behaviors for individuals who are providing unpaid care, those with social isolation or loneliness, and younger individuals, may be different, and approaches to care management with these individuals need to be proactive [38, 41, 43, 45]. Future research will involve assessing the open ended responses of participants who noted they were unable to access care during the time of COVID-19 to identify different individual, system, and public health responses that may have impacted access to healthcare and inform improvements in responsiveness for equitable future public health crises.

## Data Availability

The datasets generated and analysed during the current study are not publicly available but can be made available from the corresponding author, AP, on reasonable request.
